# Automated notifications improve time to anesthesia induction: Integrating health information technology systems to enhance perioperative efficiency

**DOI:** 10.15761/JAA.1000116

**Published:** 2018-10-03

**Authors:** Cindy Yeoh, Jennifer Mascarenhas, Kay See Tan, Luis Tollinche

**Affiliations:** Department of Anesthesiology and Critical Care Medicine, Memorial Sloan Kettering Cancer Center, USA

## Abstract

**Objective:**

To evaluate the effects of health information technology systems integration on perioperative efficiency by investigating if automated notifications of patient arrival to the operating room leads to decreased time to induction by anesthesiologists.

**Methods:**

We performed a retrospective chart review of all outpatient and short-stay patients who received General Anesthesia at our institution between July 1, 2017 and June 30, 2018.

Time was used as a measure of efficiency between the two comparison groups.

The two comparison groups were as follows:

Group 1: Pre-event notification implementation (July 1, 2017-Dec 31, 2017)

Group 2: Post-event notification implementation (Jan 1, 2018 – June 30, 2018)

In this study, our primary outcome measure duration (DUR) was collected from patient electronic medical records:

DUR: Time (duration in minutes) between anesthesia start and induction of anesthesia, exclusively for first case of the day.

**Results:**

Duration of induction was significantly shorter post-event notification implementation compared to pre-event implementation (median duration, 6 min vs 7 min; *p*=0.001).

**Conclusion:**

We demonstrate that health information technology systems integration improves perioperative efficiency of anesthesiologists at our institution. Further investigation is warranted to provide data to support provider buy-in and greater uptake and implementation of these systems to enhance patient care and coordination in the healthcare setting.

## Introduction

Health information technology (IT) systems have evolved to highly sophisticated levels in recent years. Coordination of care for patients is imperative in modern medicine, and we can now leverage health IT systems such as the electronic medical record and other patient and provider-centered technologies to improve upon patient care and coordination in our healthcare system.

Despite advancements in technology and software application, operating rooms continue to struggle with workflow efficiency contributing to rising patient census levels and increasing healthcare costs[1]. Healthcare facilities in the United States use a variety of advanced technologies with the goal of improving efficiency and workflow[2,3]. Improving operating room efficiency decreases turnover times and delays which translates to decreased patient wait times, improved patient experience, and decreased use of valuable OR time and resources[4]. The concentrated amount of highly specialized physicians and state of the art equipment and technology in current operating rooms renders the OR as the most expensive unit in a hospital with each minute of OR time valued between $30-80 [5–7].

Real-time locating system (RTLS) is a technology that provides immediate or real-time tracking and management of medical equipment, staff and patients within all types of healthcare settings [8]. Originally used to track and locate medical equipment, RTLS technology provides and stores location and time-specific data such as when a patient enters the pre-surgical area, when a nurse or physician interacts with a patient, and when a patient enters the operating room [9–11]. Alarm management and event notification technologies in medicine are designed to send alerts to designated healthcare workers when specific events occur. These systems enable healthcare organizations to integrate existing technologies to further improve upon workflow efficiency.

RTLS was first implemented with the goal of facilitating perioperative efficiency and workflow at our free standing ambulatory surgical center. Because our institution’s Main Campus does not have RTLS technology, we initially investigated if there was a difference in efficiency between anesthesiologists who have access to RTLS and anesthesiologists who do not. We found a small, albeit statistically significant, difference between the two groups of anesthesiologists with respect to anesthesia start times.

At our institution, nurse anesthetists and anesthesia residents accept the patient in the operating room in handoff from presurgical staff. This time point is defined as the “start of anesthesia” in the operating room. As part of our workflow, routine pre-induction procedures--placing monitors, securing IV connections, and preoxygenation--are initiated before the CRNA/resident calls the attending anesthesiologist for “induction of anesthesia”.

As of January 2018, further integration of RTLS and alert notification systems enabled text notifications that alerted the attending anesthesiologist when a patient entered the vicinity of the OR suite. Patient arrival in the OR suite now triggers an integrated RTLS notification system and a text message “Patient on OR floor” is sent to the attending anesthesiologist.

The goal of these systems integration is to improve OR efficiency by automating OR workflow and decreasing anesthesiologist response times. In this study, we seek to determine if systems integration has any effect on the perioperative efficiency of anesthesiologists. Specifically, we aimed to investigate if the automated “patient arrival to OR” notification decreased “time to induction” by anesthesiologists.

## Methods

We performed a retrospective chart review of all outpatient and short-stay patients who received General Anesthesia (GA) at our institution between July 1, 2017 and June 30, 2018. Only first cases of the day for all anesthesiologists were included in this study. Other inclusion criteria were patients over the age of 18 years, with ASA physical status classification of 1–3. ASA 4 and 5 patients were excluded to eliminate the possibility that higher acuity patients may have a longer pre-induction time (“anesthesia start to anesthesia induction time”). Duration between anesthesia start time and initiation of induction was used as a primary measure of efficiency between the two comparison groups.

The two comparison groups were as follows:

Group 1: Pre-event notification implementation (cases between July 1, 2017-Dec 31, 2017)

Group 2: Post-event notification implementation (cases between Jan 1, 2018 – June 30, 2018)

In this study, our primary outcome measure duration (DUR) was collected from patient electronic medical records:

DUR: Time (duration in minutes) between start and induction of anesthesia, exclusively for first cases of the day.

The primary outcome was compared between the two groups using Wilcoxon rank sum test. Analyses were conducted with Stata 13.1 (StataCorp, College Station, TX). All statistical tests were two-sided and *p* <0.05 is considered significant.

## Results

Through retrospective chart review of electronic patient records, 1579 records matched inclusion criteria of GA cases for patients over 18 years that were first case of the day (all case types for all surgical subspecialties) pre-event notification implementation (N=758) and post-event notification (N=821). Of these, 694 were outpatient versus 885 short stay cases. ASA classifications for all included cases were: ASA 1 (N=31), ASA 2 (N=960), and ASA 3 (N=588).

For our primary outcome measure, the duration between anesthesia start time and initiation of induction was shorter in the post-implementation group compared to the pre-implementation group; specifically, the median (25^th^, 75^th^ percentile) of the duration was 7.0 minutes (5.0, 9.0) pre-event notification implementation vs 6.0 minutes (5.0, 8.0) post-event notification (*p* =0.001, Table 1).

## Discussion

In our prior studies, we demonstrated that anesthesiologists at our institution with access to RTLS were significantly more efficient in their perioperative workflow (*p* <0.0001) than those without access to RTLS[12,13]. While recognizing several confounding factors, we concluded that access to RTLS improves perioperative efficiency of anesthesiologists in two ways:

1) It provides OR staff the ability to track the location of an entire OR team, including the patient, in real-time, which may allow for a more efficient process in deeming an OR “ready”.
2) The awareness that one is being tracked and monitored may motivate all OR staff, including anesthesiologists, to be readily available once the OR is deemed “ready”[14].

Previously, members of the OR staff, such as OR nurses and certified registered nurse anesthetists (CRNAs), would utilize RTLS by determining the location of the anesthesiologist on the computer application and notify them when the patient is on route to the OR. With the integration of RTLS with event notification systems, perioperative efficiency is further improved by workflow automation and decreased burden of OR workload. The anesthesiologist is now text paged automatically when a patient is located by RTLS as entering the OR vicinity. This permits the anesthesiologist to respond by showing up to the OR in a more immediate and timely manner.

This text message notification facilitates workflow and improves efficiency in two ways:

1) The CRNA/resident is unencumbered by the automatic notification system. They can now provide undivided attention to the patient during the preinduction period and are freed from the need to call or page the attending anesthesiologist.
2) Previously, a delay would result if the attending who was called for induction was engaged in other uninterruptable patient care activities. With the additional advanced notice of “patient arrival to OR”, the attending is now able to make better triage decisions about timing of inductions in various rooms, or engaging in patient care activities.

While the concept and application of clinical alerting systems and its integration is not novel, studies demonstrating effects of these systems on improving healthcare provider efficiency and patient care are lacking. Applications range from alert notification of critical lab values to Code Blue status. However, as integrated event notifications become more prevalent in clinical systems, we need to caution against “over-monitoring” and ineffective applications. The proliferation of platforms, software, and tools allow for the monitoring of varied events simultaneously, but it is of essence to avoid alert fatigue in the healthcare provider[15]. Hence, alert management should be selective, and application should be based on clinical relevance and level of urgency. Implementation and use should be supported by evidence-based studies demonstrating improvement in patient care. Results from our study support the use and integration of these IT systems to enhance perioperative efficiency.

Limitations from our study include the relative short duration of the study period. In aggregate, post implementation data was captured for six months. It is reasonable to question if these improvements will be sustainable or if they reflect short lived effects that are subject to human behavioral modifications. Future studies will explore whether these time efficiencies are maintained in the long term.

Additionally, given the single center, specialized cancer-focus of our institution, it is not possible to generalize our findings to all institutions who wish to implement RTLS. It is possible that a confounder or unknown bias influences our observations that would not affect other institutions. This too, should prompt future studies and warrants a larger, multi-institutional design to reproduce results.

## Conclusion

IT systems such as real-time locating and event notification systems hold great promise for enhancing the care and coordination of patients. While these systems are well-developed and widely available for adoption and use, strategies need to be implemented to target non-adopters and late adopters.

There is limited data demonstrating the efficacy of these IT systems and integration strategies in improving efficiency, care, and coordination in the healthcare setting. Further studies focused on evaluating the usefulness and benefits of these IT systems can facilitate provider buy-in and promote greater uptake of these patient-centered technologies.

## Figures and Tables

**Table 1.** Duration (in minutes) between start and induction of anesthesia

	Overall N=1579	Pre-implementation (N=758; 48%)	Post-implementation (N=821; 52%)	*p*-value
DUR	7.0 (5.0, 9.0)	7.0 (5.0, 9.0)	6.0 (5.0, 8.0)	0.001

DUR: Duration (in minutes) between start of anesthesia and initiation of induction by the attending anesthesiologist. Values are presented as median (25 ^th^, 75 ^th^ percentile).
